# Effects of High-Intensity Interval Versus Moderate-Intensity Continuous Training in Adults with Prediabetes: A Single-Blind Randomized Controlled Trial

**DOI:** 10.3390/jcm15187277

**Published:** 2026-09-19

**Authors:** Emine Ahsen Şenol, Yasin Yurt, Mehtap Malkoç, Sedef Delibaş

**Affiliations:** 1 Department of Physiotherapy and Rehabilitation, Faculty of Health Sciences, University of Kyrenia, Northern Cyprus, Mersin 10, Türkiye; 2 Department of Physiotherapy and Rehabilitation, Faculty of Health Sciences, Eastern Mediterranean University, Northern Cyprus, Mersin 10, Türkiye; yasin.yurt@emu.edu.tr (Y.Y.); mehtap.malkoc@emu.edu.tr (M.M.); 3 Faculty of Medicine, University of Kyrenia, Northern Cyprus, Mersin 10, Türkiye; sedefdelibas.ince@med.kyrenia.edu.tr

**Keywords:** prediabetes, high-intensity interval training, moderate-intensity continuous Training, glycemic control, randomized controlled trial

## Abstract

**Background:** Prediabetes is associated with impaired glycemic regulation and increased cardiometabolic risk, making effective lifestyle management important for preventing progression to type 2 diabetes. Exercise is a key component of prediabetes management, but the comparative effects of high-intensity interval training (HIIT) and moderate-intensity continuous training (MICT) remain unclear. **Methods:** This single-blind randomized controlled trial included 24 sedentary adults with prediabetes (HbA1c 5.7–6.4%; BMI > 24.9 kg/m^2^), randomized to HIIT (*n* = 12) or MICT (*n* = 12). Participants completed supervised treadmill-based aerobic exercise three times weekly for 12 weeks. The primary outcome was HbA1c, while secondary outcomes included anthropometric measures, lipid parameters, and inflammatory and metabolic biomarkers. Outcomes were evaluated using two-way repeated-measures ANOVA. **Results:** For the primary outcome, HbA1c decreased significantly over time in both groups (*p* = 0.001), with no significant group × time interaction (*p* > 0.05). Among the secondary outcomes, both interventions significantly improved body mass index, waist circumference, hip circumference, and waist-to-height ratio (all *p* ≤ 0.001), while lipid parameters remained unchanged (*p* > 0.05). Exploratory biomarker analyses showed within-group decreases in TNF-α in the HIIT group and resistin in the MICT group, an increase in osteocalcin in the MICT group, and increases in IL-6 in both groups (*p* < 0.05); however, no significant group × time interactions were observed for these biomarkers. CRP, irisin, adiponectin, and chemerin remained unchanged. **Conclusions:** Both HIIT and MICT were associated with improvements in glycemic control and anthropometric outcomes in this sample of adults with prediabetes, with no evidence of superiority of either exercise protocol. Given the small sample size and the absence of significant group × time interactions, the exploratory biomarker findings should be interpreted cautiously. These findings suggest that both exercise modalities may represent feasible options within individualized lifestyle management for adults with prediabetes. Larger studies with longer intervention periods and post-intervention follow-up are needed to clarify the comparative and longer-term effects of these exercise modalities.

## 1. Introduction

Prediabetes is a metabolic condition characterized by fasting plasma glucose or HbA1c levels above the normal range but below the diagnostic thresholds for type 2 diabetes [1]. Beyond impaired glycemic regulation, prediabetes is increasingly recognized as a state of low-grade chronic inflammation characterized by insulin resistance and alterations in metabolic and inflammatory mediators that may contribute to disease progression [2]. These abnormalities extend beyond glycemic impairment, as concomitant dyslipidemia, low-grade inflammation, and increased cardiometabolic risk contribute substantially to morbidity and mortality [3]. Elevated levels of inflammatory mediators, including C-reactive protein (CRP), interleukin-6 (IL-6), and tumor necrosis factor-α (TNF-α), may impair insulin signaling and exacerbate metabolic dysfunction. This bidirectional relationship between inflammation and glycemic dysregulation plays an important role in the progression from prediabetes to type 2 diabetes [4]. Prediabetes represents a substantial and growing global health burden, with its prevalence projected to increase further in the coming decades [5].

Lifestyle interventions are recommended as a first-line approach for the management of prediabetes, with regular physical activity and structured exercise representing key components of these interventions [6]. Exercise may improve glucose homeostasis through several complementary metabolic mechanisms, including increased insulin sensitivity, enhanced skeletal muscle glucose uptake, and reduced hepatic glucose production [7]. Current clinical guidelines support the role of exercise in improving glycemic control, including HbA1c levels and glucose tolerance [8]. However, evidence regarding the relative effectiveness of different exercise modalities on metabolic and inflammatory responses in individuals with prediabetes remains limited, and no definitive conclusions have been reached [9].

Beyond exercise prescription, comprehensive lifestyle management also depends on sustained behavioral engagement and appropriate professional support. Evidence from diabetes care highlights the potential value of multidisciplinary coordination, health education, patient–provider communication, and ongoing professional support in promoting effective self-management [10,11]. Community-based and nurse-led educational interventions have also demonstrated beneficial effects on glycemic management in individuals with diabetes [12]. These findings provide a broader clinical perspective supporting the integration of structured exercise into individualized lifestyle modification and coordinated healthcare.

In recent years, high-intensity interval training (HIIT) has received increasing attention because of its time efficiency and potential to induce substantial metabolic adaptations within relatively short periods [13]. Nevertheless, moderate-intensity continuous training (MICT) remains a conventional exercise approach widely recommended in clinical practice because of its effectiveness in improving glucose metabolism, its safety, and its potential for long-term adherence among individuals with obesity and prediabetes [9].

Exercise is a fundamental component of lifestyle interventions for the management of prediabetes and type 2 diabetes. In this context, HIIT and MICT are among the most frequently investigated exercise modalities because of their effects on metabolic control and cardiometabolic risk factors [14]. Previous studies have shown that both exercise modalities can reduce HbA1c levels in individuals with type 2 diabetes and obesity and may also improve lipid parameters, including HDL, LDL, total cholesterol, and triglycerides [15]. In addition, HIIT and MICT have been reported to exert favorable effects on inflammatory biomarkers, particularly CRP, potentially contributing to reductions in systemic inflammation and improvements in cardiometabolic risk profiles [16].

The effects of exercise may extend beyond changes in energy balance and glucose metabolism and involve the endocrine functions of skeletal muscle and adipose tissue. In particular, myokines secreted by skeletal muscle and adipokines secreted by adipose tissue play important roles in regulating metabolic homeostasis [17]. Previous studies have shown that exercise can alter the levels of metabolically relevant mediators, including irisin, adiponectin, and osteocalcin, in individuals with type 2 diabetes and obesity. Exercise may also contribute to metabolic homeostasis and the regulation of the inflammatory response by modulating inflammatory cytokines such as IL-6 and TNF-α, as well as adipokines such as resistin and chemerin [18]. Changes in these biomarkers may be associated with improvements in insulin sensitivity, glucose homeostasis, and inflammatory responses. Furthermore, comparative studies in individuals with metabolic syndrome, overweight, and obesity suggest that HIIT may induce more pronounced anti-inflammatory cytokine and myokine responses, potentially resulting in greater reductions in systemic inflammation and enhanced metabolic adaptations [19].

Although HIIT and MICT have been investigated in populations with cardiometabolic disorders, evidence directly comparing these exercise modalities in adults with prediabetes remains relatively limited [7,9]. Recent randomized evidence suggests that both HIIT and MICT may improve glycemic and cardiometabolic outcomes in individuals with prediabetes; however, their comparative effects across a broader range of metabolic and inflammatory biomarkers remain insufficiently characterized [9]. Previous exercise trials in prediabetes have primarily focused on glycemic outcomes or specific exercise protocols, leaving uncertainty about whether exercise intensity differentially influences glycemic, anthropometric, metabolic, and inflammatory responses [9,20]. Therefore, the primary objective of this randomized controlled trial was to compare the effects of 12 weeks of HIIT and MICT on HbA1c in adults with prediabetes. The secondary objectives were to compare the effects of these exercise modalities on lipid profiles, metabolic and inflammatory biomarkers, anthropometric measures, body composition, and cardiometabolic risk indicators.

## 2. Materials and Methods

### 2.1. Study Design

**Figure 1:**
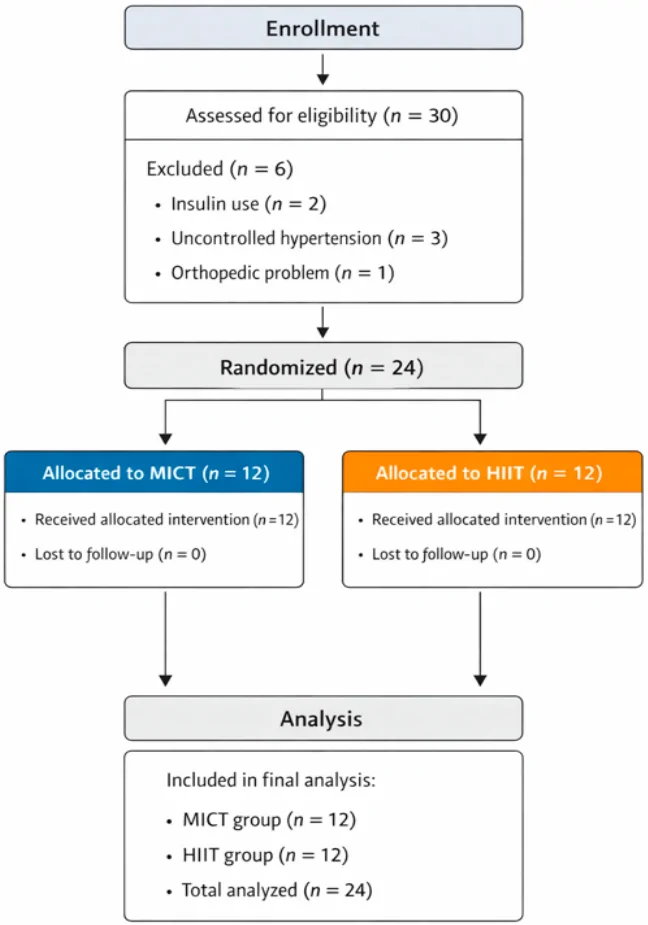
CONSORT flow diagram of participant recruitment, allocation and analysis.

This study was conducted as a single-blind randomized controlled trial comparing the effects of HIIT and MICT in adults with prediabetes. The trial was registered on ClinicalTrials.gov (NCT06831266) and approved by the Eastern Mediterranean University Scientific Research and Publication Ethics Board (ETK00-2023-0184). Written informed consent was obtained from all participants before study participation (Figure 1). The trial record was initially released on 29 January 2025, before the study commenced on 19 February 2025, and the study was completed on 30 November 2025. No changes were made to the planned intervention protocols after trial commencement. The exercise interventions were conducted at the Physiotherapy and Rehabilitation Laboratory, Faculty of Health Sciences, University of Kyrenia, Kyrenia, Mersin 10, Türkiye. Biochemical analyses of blood samples were performed at the Biochemistry Laboratory, Faculty of Medicine, Near East University, Nicosia, Mersin 10, Türkiye. The CONSORT 2025 Checklist is provided in Supplementary File S1.

### 2.2. Participants

Participants were recruited from the community on a voluntary basis. Individuals aged 18–60 years with HbA1c levels of 5.7–6.4%, a body mass index (BMI) > 24.9 kg/m^2^, a sedentary lifestyle, and no ongoing dietary program were eligible for inclusion. Exclusion criteria were the use of insulin or insulin-derived medications, hypertension that remained uncontrolled despite pharmacological treatment, a history of heart disease or myocardial infarction, a history of stroke, and lower-extremity orthopedic conditions that could interfere with participation in the exercise interventions.

### 2.3. Sample Size Calculation

The sample size was determined based on data from the study by RezkAllah and Takla [20]. An a priori power analysis was performed using G*Power version 3.1.9.2 (Universität Düsseldorf, Düsseldorf, Germany), with an effect size of d = 1.976, a statistical power of 80% (1 − β), and a significance level of 5% (α = 0.05). The analysis indicated that a minimum of nine participants per group was required. To account for potential dropouts, the calculated sample size was increased by 20%, resulting in a target sample size of 12 participants per group and a total sample size of 24 participants.

### 2.4. Randomization & Blinding

A total of 24 participants included in the study were randomly allocated to the two exercise intervention groups in a 1:1 ratio using a computer-generated randomization sequence [21]. The randomization list was prepared by an independent researcher who was not involved in participant enrollment, assessment, or the intervention process. To ensure allocation concealment, each group assignment was placed in an opaque, light-impermeable, sequentially numbered, sealed envelope. After participants met the eligibility criteria and provided informed consent, the next envelope in sequence was opened and the participant was assigned to the corresponding intervention group. Clinical assessments and biochemical measurements were performed by researchers who were blinded to participants’ group assignments to minimize the risk of assessment bias. Assessments were performed under standardized conditions before the intervention and at the end of the 12-week intervention period.

### 2.5. Primary Outcome

HbA1c was defined as the primary outcome of the study and was assessed as an indicator of glycemic control over the preceding 8–12 weeks. For HbA1c measurement, venous blood samples were collected from the antecubital vein into EDTA-containing tubes. HbA1c levels were measured using NGSP-certified automated analyzers based on high-performance liquid chromatography (HPLC) and traceable to the International Federation of Clinical Chemistry and Laboratory Medicine (IFCC) reference system (Alinity c4000, Abbott GmbH & Co. KG, Wiesbaden, Germany). Results were reported in NGSP units (%). Measurements were performed at baseline and after the 12-week intervention using the same laboratory, equipment, and analytical protocol [22].

### 2.6. Secondary Outcomes

Secondary outcomes included lipid profile, metabolic and inflammatory biomarkers, anthropometric measures, body composition, and cardiometabolic risk indicators. The biochemical outcomes comprised high-density lipoprotein cholesterol (HDL), low-density lipoprotein cholesterol (LDL), total cholesterol, triglycerides, C-reactive protein (CRP), irisin, adiponectin, resistin, chemerin, osteocalcin, interleukin-6 (IL-6), and tumor necrosis factor-α (TNF-α) [23].

Blood samples were collected from the antecubital vein in the morning after an overnight fast of 8–12 h. Samples were centrifuged, and the separated serum was stored at −80 °C until analysis in accordance with the manufacturers’ recommendations [24]. Serum total cholesterol, HDL cholesterol, and triglyceride levels were determined using enzymatic colorimetric methods (Alinity c4000, Abbott GmbH & Co. KG, Wiesbaden, Germany), whereas LDL cholesterol was calculated using the Friedewald formula. CRP levels were analyzed using a high-sensitivity immunoturbidimetric method. Serum irisin, adiponectin, resistin, chemerin, osteocalcin, IL-6, and TNF-α levels were measured using enzyme-linked immunosorbent assay (ELISA) (FineTest, Wuhan Fine Biotech Co., Ltd., Wuhan, China) in accordance with the manufacturers’ protocols [25]. All biochemical analyses were performed under the same laboratory conditions using the same equipment and kit lots. ELISA data were analyzed using SoftMax Pro software (Molecular Devices, San Jose, CA, USA). Measurements were performed at baseline and after the 12-week intervention using the same protocol.

Anthropometric measures and parameters reflecting body fat distribution were assessed as secondary outcomes to evaluate changes in cardiometabolic risk and body composition. All measurements were performed by the same physiotherapist using a standardized protocol. Waist circumference, hip circumference, waist-to-hip ratio (WHR), waist-to-height ratio (WHtR), A Body Shape Index (ABSI), and Body Adiposity Index (BAI) were assessed. Waist circumference was measured at the midpoint between the lowest rib and the iliac crest while participants were standing and breathing normally. Hip circumference was measured at the widest point of the gluteal region. WHR was calculated by dividing waist circumference by hip circumference, whereas WHtR was calculated by dividing waist circumference by height [26]. ABSI was calculated according to the formula described in the literature using waist circumference, BMI, and height [27]. BAI was calculated based on hip circumference and height and was included as an indirect indicator of total body adiposity [28].

Body weight, height, and body mass index (BMI) were assessed as measures of body composition. Body weight was measured in kilograms using a calibrated digital scale, and height was measured in meters using a stadiometer. BMI was calculated as body weight in kilograms divided by height in meters squared (kg/m^2^) [29].

Subcutaneous adipose tissue was assessed using skinfold thickness measurements performed with a skinfold caliper (SH5020, Saehan Corporation, Changwon, Republic of Korea). Measurements were obtained on the right side of the body using anatomical reference points defined in the literature for each site. Skinfold thickness was measured at the subscapular, biceps, triceps, chest, midaxillary, suprailiac, abdominal, thigh, and leg regions [30].

Each anthropometric and body composition measurement was performed three times, and the mean value was used in the analyses. All measurements were performed under the same standardized conditions at baseline and after the 12-week intervention.

### 2.7. Exercise Protocols (HIIT vs. MICT)

Participants completed a structured 12-week aerobic exercise program according to their assigned intervention group. All exercise sessions were performed three times per week on a treadmill (Vertex Home Pro, Model 520S; Yongkang Dafang Industry and Trade Co., Ltd., Yongkang city, China) under the continuous supervision of a physiotherapist, with exercise intensity prescribed according to each participant’s peak heart rate (PHR). PHR was determined from a maximal exercise test using the Bruce protocol at baseline (T2100 treadmill, GE Healthcare, Waukesha, WI, USA; CASE™ exercise testing software, version 6.7, GE Healthcare, Milwaukee, WI, USA). Exercise intensity was monitored using a heart rate monitor throughout each session (Polar H10, Polar Electro Oy, Kempele, Finland). All sessions included standardized warm-up and cool-down periods, and participants were monitored by the physiotherapist for adverse events or exercise-related symptoms. Participants were instructed to maintain their usual lifestyle throughout the 12-week intervention period and to avoid intentional changes to their habitual diet or physical activity outside the prescribed exercise program. All participants completed the 36 scheduled exercise sessions, corresponding to 100% adherence to the prescribed exercise program. The progressive 12-week HIIT and MICT exercise protocols are presented in Table 1 [20,31].

### 2.8. Statistical Analysis

Continuous variables were summarized as mean ± standard deviation (SD), whereas categorical variables were summarized as frequencies and percentages. The normality of continuous variables was assessed using the Shapiro–Wilk test, and homogeneity of variances was evaluated using Levene’s test. Baseline categorical characteristics were compared between groups using the chi-square test or Fisher’s exact test, as appropriate.

Continuous outcome variables were analyzed using a two-way mixed-design repeated-measures analysis of variance (RM-ANOVA), with group (HIIT vs. MICT) as the between-subjects factor and time (baseline vs. post-intervention) as the within-subjects factor. The group × time interaction was used to evaluate whether changes over time differed between the intervention groups. Type III sums of squares were used. Partial eta-squared (η^2^_p_) was reported as a measure of effect size. Follow-up comparisons were performed using the Holm–Sidak method to evaluate within-group changes from baseline to post-intervention and between-group differences at each time point.

For TNF-α, outlier screening was performed using the Grubbs test and standardized z-scores. One participant in the HIIT group was identified as a statistical outlier at both time points (Grubbs *p* < 0.001; |z| > 3.0) and was therefore excluded from the TNF-α analysis. All other analyses were performed using the full sample.

All statistical tests were two-tailed, and a *p*-value < 0.05 was considered statistically significant. All statistical analyses were performed using R software (version 4.4.2; R Core Team, R Foundation for Statistical Computing, Vienna, Austria). There were no missing outcome data; therefore, no imputation procedures were required.

## 3. Results

### Participant Enrolment and Baseline Characteristics

A total of 24 participants were randomized to the HIIT (*n* = 12) and MICT (*n* = 12) groups. All randomized participants completed the 12-week intervention and were included in the final analyses, with no dropouts in either group. No adverse events were reported during the intervention period. Baseline demographic and anthropometric characteristics were generally similar between the HIIT and MICT groups.

For the primary outcome, HbA1c levels decreased significantly from baseline to post-intervention in both the HIIT and MICT groups (both *p* = 0.001). However, the group × time interaction was not statistically significant (*p* = 0.392; η^2^_p_ = 0.03), indicating no significant difference in the change in HbA1c between the two groups (Table 2).

For lipid profile outcomes, no significant within-group changes were observed in HDL, LDL, total cholesterol, or triglyceride levels in either group (all *p* > 0.05). Furthermore, no significant group × time interactions were observed for any lipid parameter (all *p* > 0.05) (Table 2).

When anthropometric parameters were examined, within-group analyses showed statistically significant reductions from baseline in body mass index (BMI), waist circumference, hip circumference, waist-to-height ratio (WHtR), Body Adiposity Index (BAI), and A Body Shape Index (ABSI) in both the HIIT and MICT groups (all *p* ≤ 0.001 in the HIIT group; *p* = 0.017 for ABSI and *p* ≤ 0.001 for all other parameters in the MICT group). In contrast, waist-to-hip ratio (WHR) did not change significantly in either group (HIIT: *p* = 0.109; MICT: *p* = 0.373). Regarding the group × time interaction, no statistically significant interaction was detected for any of the anthropometric parameters (all *p* > 0.05) (Table 3).

For inflammatory and metabolic biomarkers, CRP levels did not change significantly in either the HIIT or MICT group (*p* = 0.329 and *p* = 0.772, respectively). TNF-α levels decreased significantly within the HIIT group (*p* = 0.047), whereas the change in the MICT group was not significant (*p* = 0.298). IL-6 levels increased significantly in both the HIIT (*p* = 0.046) and MICT (*p* = 0.001) groups. No significant within-group changes were observed in irisin, adiponectin, or chemerin levels (all *p* > 0.05). Resistin levels decreased significantly in the MICT group (*p* = 0.037) but not in the HIIT group (*p* = 0.084), whereas osteocalcin levels increased significantly in the MICT group (*p* = 0.005) but not in the HIIT group (*p* = 0.163). No statistically significant group × time interactions were observed for any of the inflammatory or metabolic biomarkers (all *p* > 0.05) (Table 4).

## 4. Discussion

In this randomized controlled trial, 12 weeks of supervised HIIT and MICT resulted in significant improvements in HbA1c and several anthropometric outcomes in adults with prediabetes, whereas no significant group × time interactions were observed, indicating no evidence of superiority of either exercise modality for these outcomes. In contrast, lipid parameters did not change significantly following either intervention, while selected inflammatory and metabolic biomarkers showed within-group changes that should be interpreted cautiously given the small sample size, the number of biomarkers examined, and the absence of significant group × time interactions. Overall, these findings support the potential value of both HIIT and MICT as exercise-based strategies for improving glycemic control and anthropometric risk indicators in adults with prediabetes, while suggesting that their effects on lipid and biomarker outcomes may be less consistent over a 12-week intervention period.

The significant reduction in HbA1c observed following both HIIT and MICT is consistent with previous evidence supporting the beneficial effects of structured aerobic exercise on glycemic control in individuals with prediabetes and related glucose metabolism impairments [9,32,33]. Importantly, the absence of a significant group × time interaction in the present study indicates that neither exercise modality demonstrated superiority for improving HbA1c over the 12-week intervention period. Previous comparative evidence has not been entirely uniform in this regard; while both HIIT and MICT have been associated with improvements in glycemic regulation, some studies and meta-analyses have suggested greater benefits with HIIT for selected glycemic outcomes [32,33]. Differences in study populations, exercise protocols, intervention duration, and baseline metabolic status may contribute to variability across studies. From a clinical perspective, the present findings suggest that both exercise modalities may represent reasonable options for structured aerobic exercise in adults with prediabetes, without assuming a clear glycemic advantage of one modality over the other.

The improvement in HbA1c observed following both exercise interventions may be partly explained by established physiological adaptations to exercise. Regular exercise can enhance skeletal muscle glucose uptake through insulin-independent pathways, including increased GLUT4 translocation, while also promoting mitochondrial oxidative capacity and peripheral insulin sensitivity [34]. Although these mechanisms were not directly assessed in the present study, they provide plausible physiological explanations for the improvements in glycemic control observed following both exercise protocols.

The improvements observed in several anthropometric measures are consistent with evidence showing that both HIIT and MICT can favorably influence body composition and adiposity-related outcomes [9,35,36,37]. Previous systematic reviews and meta-analyses have reported reductions in body adiposity following both exercise modalities, although the relative magnitude of these effects may vary according to the population, training protocol, and outcome assessed [35,36,37]. In the present study, the absence of significant group × time interactions across the anthropometric outcomes provides no evidence of a differential effect of HIIT and MICT over the 12-week intervention period. These changes may be clinically relevant in adults with prediabetes, particularly because measures of central adiposity are closely related to cardiometabolic risk; however, the findings should be interpreted in the context of the small sample size and relatively short intervention period.

Beyond physiological responses, the clinical applicability of an exercise program may also depend on factors such as time requirements, individual preferences, tolerance, adherence, and long-term sustainability. Although HIIT has been proposed as a time-efficient exercise strategy, the choice between HIIT and MICT should not be based solely on the expectation of superior metabolic effects [7,9]. In the absence of clear evidence of superiority in the present study, individualized exercise prescription that considers patient preferences, functional capacity, and the likelihood of sustained participation may represent a more appropriate approach for adults with prediabetes.

In contrast to the improvements in glycemic and anthropometric outcomes, neither HIIT nor MICT produced significant changes in HDL, LDL, total cholesterol, or triglyceride levels over the 12-week intervention period. This finding is consistent with evidence indicating that changes in blood lipid concentrations following exercise are variable and may depend on factors such as exercise volume and intensity, intervention duration, baseline metabolic status, and other lifestyle-related factors [38,39]. Previous systematic reviews and meta-analyses have similarly reported heterogeneous lipid responses to HIIT and MICT, with no consistent evidence that one modality is superior to the other for modifying blood lipid concentrations [38,40]. Therefore, the absence of significant lipid changes in the present study should not necessarily be interpreted as a lack of metabolic benefit, but rather may reflect differences in the responsiveness and time course of glycemic and lipid-related adaptations to exercise.

Several factors may help explain the absence of significant changes in lipid parameters despite improvements in glycemic and anthropometric outcomes. Lipid responses to exercise are influenced by complex and interacting determinants, and exercise-induced adaptations in lipid metabolism may involve changes in lipolysis, fatty acid utilization, and the regulation of lipid handling across multiple tissues [41]. These mechanisms may respond differently according to exercise dose, intervention duration, baseline metabolic status, and accompanying lifestyle factors. Importantly, these potential mechanisms were not directly assessed in the present study and should therefore be regarded as possible explanations rather than demonstrated mechanisms. In a broader context, susceptibility to lipid and metabolic dysfunction may also reflect long-term determinants extending beyond adult lifestyle. Emerging evidence suggests that disturbances in maternal lipid metabolism and oxidative stress during pregnancy may influence fetal metabolic programming and potentially contribute to metabolic susceptibility later in life [42]. Although developmental factors were not assessed in the present study and cannot explain the observed lipid response directly, this perspective highlights the complex and potentially life-course nature of lipid dysregulation.

The inflammatory biomarker findings require cautious interpretation. Although TNF-α decreased significantly within the HIIT group and IL-6 increased significantly within both exercise groups, no significant group × time interactions were observed for these markers. Therefore, the within-group reduction in TNF-α should not be interpreted as evidence of a specific anti-inflammatory advantage of HIIT over MICT. Previous systematic reviews and meta-analyses have reported that exercise may influence inflammatory biomarkers in individuals with metabolic disorders, although the magnitude and direction of these responses vary according to the population, exercise protocol, biomarker assessed, and timing of measurement [19,43]. In the present study, the small sample size and the number of biomarkers examined further warrant caution when interpreting these individual within-group findings.

The significant increase in IL-6 observed within both exercise groups warrants careful interpretation. IL-6 has complex biological functions and may act not only as a pro-inflammatory cytokine but also as an exercise-responsive myokine involved in metabolic and inflammatory signaling [44]. However, because the present study did not assess the acute IL-6 response to individual exercise sessions or determine the tissue source of circulating IL-6, the observed post-intervention increase cannot be directly attributed to skeletal muscle-derived IL-6. Furthermore, the absence of a significant group × time interaction indicates that the IL-6 response did not differ significantly between HIIT and MICT. CRP levels, in contrast, did not change significantly within either group, and no significant group × time interaction was observed. The lack of a detectable CRP response may reflect the variability of inflammatory adaptations to exercise and the influence of factors such as baseline inflammatory status, intervention duration, exercise dose, and individual metabolic characteristics [43,45]. Overall, these findings emphasize the complexity of inflammatory biomarker responses to exercise and should be interpreted cautiously in the context of the small sample size and multiple biomarker assessments.

Among the metabolic biomarkers, resistin levels decreased significantly and osteocalcin levels increased significantly within the MICT group, whereas no significant changes were observed within the HIIT group. Resistin is an adipokine implicated in metabolic and inflammatory regulation, whereas osteocalcin has been increasingly recognized for its potential role in the interaction between bone and energy metabolism [44]. Exercise-induced changes in these mediators have been reported previously; however, their responses may vary according to the population and exercise protocol [18,46]. Importantly, no significant group × time interactions were observed for either resistin or osteocalcin in the present study. Therefore, these within-group findings should be considered exploratory and should not be interpreted as evidence of a specific metabolic or hormonal advantage of MICT over HIIT.

### 4.1. Perspectives for Clinical Practice

The present findings may have practical implications for exercise prescription in adults with prediabetes. Given that no significant group × time differences were observed between HIIT and MICT for glycemic and anthropometric outcomes, exercise modality may be individualized according to functional capacity, patient preferences, feasibility, and the likelihood of sustained adherence rather than being selected on the assumption that one approach is universally superior. In clinical practice, exercise should be considered as one component of comprehensive lifestyle management, alongside nutritional guidance, health education, and strategies supporting long-term self-management [10,11,12]. Coordination among healthcare professionals may facilitate appropriate assessment, individualized exercise prescription, monitoring, health education, and adherence support [10,11,12]. Such a multidisciplinary approach may help translate structured exercise interventions into sustainable lifestyle strategies for adults with prediabetes.

### 4.2. Strengths and Limitations

The main strengths of this study include its randomized controlled, single-blind design and the direct comparison of supervised HIIT and MICT protocols. In addition, the combined assessment of glycemic, anthropometric, lipid, inflammatory, and metabolic biomarkers enabled a comprehensive evaluation of exercise-related responses across multiple cardiometabolic domains. Furthermore, all participants completed the 12-week intervention with full adherence to the prescribed exercise sessions, strengthening the internal consistency of the intervention and supporting the interpretation of the observed findings.

Several limitations should be considered when interpreting the present findings. First, the sample size was calculated based on the primary outcome of HbA1c and may therefore have provided limited statistical power to detect changes in secondary outcomes, particularly lipid and inflammatory or metabolic biomarkers. The relatively small sample size also limits the generalizability of the findings and warrants caution when interpreting individual within-group biomarker changes. Second, the 12-week intervention period may have been insufficient to detect adaptations in outcomes that may require a longer duration of exercise training, particularly lipid-related parameters. Third, the assessment of multiple inflammatory and metabolic biomarkers increases the possibility of chance findings and therefore these secondary biomarker results should be considered exploratory. In addition, dietary intake and physical activity outside the supervised exercise sessions were not controlled, which may have influenced the observed responses. Finally, the absence of a post-intervention follow-up prevents conclusions regarding the longer-term maintenance of the observed improvements.

## 5. Conclusions

This study showed that 12 weeks of supervised HIIT and MICT was associated with significant improvements in HbA1c and several anthropometric outcomes in adults with prediabetes, with no significant group × time interactions observed for these outcomes. In contrast, lipid parameters did not change significantly, while selected inflammatory and metabolic biomarkers showed within-group changes that should be interpreted cautiously. These findings suggest that both HIIT and MICT may represent feasible exercise options for adults with prediabetes, with exercise prescription individualized according to patient preferences, functional capacity, adherence, and long-term sustainability rather than an expectation of superiority of one modality over the other. Further studies with larger sample sizes, longer intervention periods, and post-intervention follow-up are needed to clarify the comparative and longer-term effects of these exercise modalities, particularly on lipid, inflammatory, and metabolic biomarker outcomes.

## Figures and Tables

**Table 1:** Exercise protocols for the HIIT and MICT groups.

	HIIT	MICT
Frequency	3 sessions/week for 12 weeks	3 sessions/week for 12 weeks
Intensity	Weeks 1–2: Exercise at 85% of PHR with active recovery at 60% of PHR (4 × 1 min) Weeks 3–7: Exercise at 90% of PHR with active recovery at 60% of PHR (6 × 1 min) Weeks 8–12: Exercise at 90% of PHR with active recovery at 60% of PHR (8 × 1 min)	Weeks 1–2: Exercise at 65% of PHR for 22 min Weeks 3–7: Exercise at 75% of PHR for 30 min Weeks 8–12: Exercise at 75% of PHR for 38 min
Duration	Weeks 1–2: 8 min/session Weeks 3–7: 12 min/session Weeks 8–12: 16 min/session Warm-up: 5 min/day (60% of PHR) Cool-down: 3 min/day (60% of PHR)	Weeks 1–2: 22 min/session Weeks 3–7: 30 min/session Weeks 8–12: 38 min/session Warm-up: 5 min/day (60% of PHR) Cool-down: 3 min/day (60% of PHR)
Type	Treadmill-based aerobic exercise	Treadmill-based aerobic exercise

Abbreviations: HIIT, high-intensity interval training; MICT, moderate-intensity continuous training; PHR, peak heart rate.

**Table 2:** Effects of HIIT and MICT on HbA1c and lipid profile outcomes over time.

Variable	Group	Baseline (Mean ± SD)	Post (Mean ± SD)	Within-Group *p*	Group × Time *p*	η^2^_p_
HbA1c (%)	HIIT	5.92 ± 0.21	5.57 ± 0.26	0.001 *	0.392	0.03
	MICT	5.88 ± 0.23	5.46 ± 0.27	0.001 *		
HDL (mg/dL)	HIIT	56.00 ± 15.61	57.33 ± 15.07	0.550	0.063	0.01
	MICT	50.08 ± 12.05	50.08 ± 11.52	1.000		
LDL (mg/dL)	HIIT	119.92 ± 36.09	131.08 ± 32.87	0.151	0.385	0.03
	MICT	127.42 ± 48.25	128.58 ± 47.29	0.895		
Total cholesterol (mg/dL)	HIIT	200.08 ± 40.35	211.92 ± 36.40	0.262	0.508	0.02
	MICT	202.17 ± 62.90	204.50 ± 56.07	0.819		
Triglycerides (mg/dL)	HIIT	121.17 ± 56.58	117.33 ± 35.24	0.804	0.595	0.01
	MICT	122.42 ± 72.62	129.08 ± 58.39	0.598		

* Data are presented as mean ± SD. Within-group comparisons were performed using Holm–Sidak-adjusted follow-up tests. Group × time interactions were assessed using two-way mixed-design repeated-measures analysis of variance (RM-ANOVA). η^2^_p_ indicates partial eta-squared as a measure of effect size. *p* < 0.05 was considered statistically significant. HIIT, high-intensity interval training; MICT, moderate-intensity continuous training; HbA1c, glycated hemoglobin; HDL, high-density lipoprotein; LDL, low-density lipoprotein; SD, standard deviation.

**Table 3:** Effects of HIIT and MICT on anthropometric outcomes over time.

Variable	Group	Baseline (Mean ± SD)	Post (Mean ± SD)	Within-Group *p*	Group × Time *p*	η^2^_p_
Body mass index (kg/m^2^)	HIIT	34.09 ± 5.44	32.61 ± 5.49	0.001 *	0.854	0.00
	MICT	35.73 ± 5.06	34.20 ± 4.77	0.001 *		
Waist circumference (cm)	HIIT	91.79 ± 10.88	85.62 ± 11.07	0.001 *	0.290	0.05
	MICT	95.58 ± 8.18	90.76 ± 8.62	0.001 *		
Hip circumference (cm)	HIIT	115.04 ± 7.67	109.08 ± 6.92	0.001 *	0.552	0.02
	MICT	117.46 ± 11.92	112.42 ± 11.58	0.001 *		
Waist-to-hip ratio	HIIT	0.80 ± 0.07	0.78 ± 0.07	0.109	0.595	0.01
	MICT	0.82 ± 0.07	0.81 ± 0.05	0.373		
Waist-to-height ratio	HIIT	0.57 ± 0.06	0.53 ± 0.06	0.001 *	0.311	0.05
	MICT	0.60 ± 0.06	0.57 ± 0.06	0.001 *		
Body Adiposity Index	HIIT	38.67 ± 4.48	35.71 ± 3.67	0.001 *	0.552	0.02
	MICT	40.23 ± 5.99	37.74 ± 5.88	0.001 *		
A Body Shape Index	HIIT	0.07 ± 0.00	0.07 ± 0.00	0.001 *	0.259	0.06
	MICT	0.07 ± 0.00	0.07 ± 0.00	0.017 *		

* Data are presented as mean ± SD. Within-group comparisons were performed using Holm–Sidak-adjusted follow-up tests. Group × time interactions were assessed using two-way mixed-design repeated-measures analysis of variance (RM-ANOVA). η^2^_p_ indicates partial eta-squared as a measure of effect size. *p* < 0.05 was considered statistically significant. HIIT, high-intensity interval training; MICT, moderate-intensity continuous training; BMI, body mass index; WHR, waist-to-hip ratio; WHtR, waist-to-height ratio; BAI, Body Adiposity Index; ABSI, A Body Shape Index; SD, standard deviation.

**Table 4:** Effects of HIIT and MICT on inflammatory and metabolic biomarkers over time.

Variable	Group	Baseline (Mean ± SD)	Post (Mean ± SD)	Within-Group *p*	Group × Time *p*	η^2^_p_
CRP (mg/L)	HIIT	1.12 ± 2.03	0.60 ± 0.92	0.329	0.317	0.05
	MICT	0.69 ± 0.61	0.79 ± 1.22	0.772		
TNF-α (pg/mL)	HIIT	97.68 ± 102.11	68.50 ± 64.50	0.047 *	0.126	0.11
	MICT	32.12 ± 47.65	25.19 ± 29.46	0.298		
IL-6 (pg/mL)	HIIT	1.49 ± 1.29	2.23 ± 1.31	0.046 *	0.292	0.05
	MICT	0.87 ± 0.65	2.14 ± 1.88	0.001 *		
Irisin (ng/mL)	HIIT	8.69 ± 3.45	8.79 ± 3.70	0.785	0.666	0.01
	MICT	6.89 ± 3.69	6.77 ± 3.89	0.735		
Adiponectin (µg/mL)	HIIT	43.35 ± 11.78	43.77 ± 17.59	0.916	0.728	0.01
	MICT	34.50 ± 20.33	36.88 ± 21.08	0.552		
Resistin (ng/mL)	HIIT	2558.33 ± 222.20	2373.83 ± 400.24	0.084	0.775	0.00
	MICT	2288.67 ± 834.65	2062.42 ± 908.80	0.037 *		
Chemerin (ng/mL)	HIIT	13.24 ± 1.34	11.97 ± 3.09	0.221	0.752	0.00
	MICT	10.27 ± 4.29	9.46 ± 5.03	0.428		
Osteocalcin (ng/mL)	HIIT	6.61 ± 4.60	8.28 ± 6.02	0.163	0.258	0.06
	MICT	4.39 ± 3.54	7.95 ± 7.13	0.005 *		

* Data are presented as mean ± SD. Within-group comparisons were performed using Holm–Sidak-adjusted follow-up tests. Group × time interactions were assessed using two-way mixed-design repeated-measures analysis of variance (RM-ANOVA). η^2^_p_ indicates partial eta-squared as a measure of effect size. *p* < 0.05 was considered statistically significant. One participant in the HIIT group was excluded from the TNF-α analysis because of an identified outlier; all other analyses included the full sample. HIIT, high-intensity interval training; MICT, moderate-intensity continuous training; CRP, C-reactive protein; TNF-α, tumor necrosis factor-alpha; IL-6, interleukin-6; SD, standard deviation.

## Data Availability

The data supporting the findings of this study are available from the corresponding author upon reasonable request, subject to ethical and privacy considerations. The study protocol and prespecified statistical analysis plan are available from the corresponding author upon reasonable request.

## Supplementary Materials

The following supporting information can be downloaded at: https://www.mdpi.com/article/10.3390/jcm15187277/s1, File S1: CONSORT 2025 Checklist.
