# Supplementary material for: Effects of High-Intensity Interval Versus Moderate-Intensity Continuous Training in Adults with Prediabetes: A Single-Blind Randomized Controlled Trial
Source: J Clin Med. 2026 Sep 19;15(18):7277. doi: 10.3390/jcm15187277 (PMC13607590; doi:10.3390/jcm15187277)
Supplement: Supplementary file 1 [file jcm-15-07277-s001.zip › jcm-4529935-supplementary.pdf]

CONSORT 2025 Checklist

Randomized Controlled Trial – Journal of Clinical Medicine Submission

Manuscript: Comparative Effects of High-Intensity Interval Training and Moderate-Intensity Continuous Training on Glycemic Control and Metabolic and Inflammatory Markers in Adults with Prediabetes: A Single-Blind Randomized Controlled Trial

Note: This checklist reflects the final manuscript prepared for submission to the Journal of Clinical Medicine. "Partial" indicates that only part of a CONSORT item is explicitly reported in the manuscript.

Item	Topic	Checklist focus	Page(s)	Status / note
1a	Title	Randomized trial identified in title	1	Reported
1b	Structured abstract	Design, methods, results and conclusions summarized	1	Reported
2	Trial registration	Registry and identifier; registration date/URL	2	Reported – ClinicalTrials.gov identifier NCT06831266 and initial release date (29 January 2025) are stated; the trial was registered before the actual study start date.
3	Protocol/SAP access	Access to trial protocol and statistical analysis plan	12	Reported – study protocol and prespecified statistical analysis plan are available from the corresponding author upon reasonable request.
4	Data sharing	Availability of de-identified data/code/materials	12	Reported in Data Availability Statement.
5a	Funding	Funding/support and role of funder	12	Reported – funding source is stated and the funder had no role in study design, data collection, data analysis or interpretation, manuscript preparation, or the decision to publish.
5b	Conflicts	Financial/other conflicts of interest	12	Reported.
6	Background/rationale	Scientific background and rationale	1–2	Reported.
7	Objectives	Specific trial objective(s)	2	Reported.
8	Patient/public involvement	Patient/public involvement in design, conduct or reporting	—	NR – patient/public involvement is not described.
9	Trial design	Design, allocation ratio and trial framework	2–3	Partial – randomized two-group design and equal allocation are described; the trial framework is not explicitly labelled.
10	Protocol changes	Important post-commencement changes and reasons	2	Reported – no changes to the planned intervention protocols were made after trial commencement.
11	Setting	Trial setting and location(s)	2	Reported – exercise interventions and biochemical analysis locations are explicitly stated.
12a	Eligibility	Participant inclusion/exclusion criteria	3	Reported.
12b	Eligibility of sites/deliverers	Criteria for sites/intervention providers, if applicable	—	NR / not clearly applicable.
13	Interventions	Replicable details of intervention and comparator	4–5	Reported; detailed HIIT and MICT protocol table included.
14	Outcomes	Prespecified primary/secondary outcomes, measurement and time point	3–4	Reported – primary and secondary outcomes, measurement procedures and baseline/post-intervention time points are described.
15	Harms assessment	How harms/unintended effects were defined and assessed	4–5	Reported – all sessions were continuously supervised by a physiotherapist who monitored participants for adverse events or exercise-related symptoms.
16a	Sample size	Sample-size calculation and assumptions	3	Reported.
16b	Interim analyses	Interim analyses/stopping rules, if applicable	—	NR / no interim analyses or stopping rules stated.
17a	Sequence generation	Who generated sequence and method used	3	Reported – sequence generated with a computer-based random number generator by an independent researcher.
17b	Randomization type	Type/restrictions of randomization	3	Reported – simple randomization; no restrictions described.
18	Allocation concealment	Mechanism used to conceal allocation	3	Reported – opaque, light-impermeable, sequentially numbered, sealed envelopes.
19	Implementation	Access of enrolling/assigning personnel to sequence	3	Reported – independent researcher prepared the randomization list; enrollment/assignment procedure is described.

Item	Topic	Checklist focus	Page(s)	Status / note
20a	Blinding	Who was blinded after allocation	3	Reported – researchers performing clinical assessments and biochemical measurements were blinded to group assignment.
20b	Blinding method	How blinding was achieved / intervention similarity	3	Partial – assessor blinding is described; additional operational detail is limited.
21a	Statistical methods	Methods for primary/secondary outcome comparisons	5	Reported – two-way mixed-design repeated-measures ANOVA and follow-up procedures are described.
21b	Analysis population	Who was included in each analysis and assigned group	5–6	Reported – all 24 randomized participants completed the intervention and were included in final analyses; TNF-α analysis excluded one statistical outlier as specified.
21c	Missing data	Handling of missing outcome data	5–6	Reported – there were no missing outcome data; therefore, no imputation procedures were required.
21d	Additional analyses	Methods for subgroup/sensitivity/other analyses	5	Reported where applicable – TNF-α outlier screening/exclusion is described; no subgroup or sensitivity analyses are reported.
22a	Participant flow	Randomized, treated and analyzed numbers by group	6	Reported – CONSORT flow diagram and text provide randomized, allocated, completed and analyzed numbers by group.
22b	Losses/exclusions	Post-randomization losses/exclusions and reasons	6	Reported – no post-randomization dropouts occurred.
23a	Recruitment/follow-up dates	Dates defining recruitment and follow-up periods	2	Partial – actual study start date (19 February 2025) and study completion date (30 November 2025) are stated; a distinct recruitment-period end date is not reported.
23b	Early stopping	Reason trial ended/stopped, if relevant	2, 6	Not applicable – trial was completed; no early stopping is reported.
24a	Intervention delivery	What was actually delivered, adherence/fidelity/provider as appropriate	4–5	Reported – all participants completed all 36 scheduled exercise sessions, corresponding to 100% adherence.
24b	Concomitant care	Other care received by each group	4–5	Reported – participants were instructed to maintain their usual lifestyle and not intentionally change habitual diet or physical activity outside the prescribed program.
25	Baseline data	Baseline demographic/clinical characteristics by group	6	Reported in text – groups are described as comparable at baseline for demographic variables.
26	Numbers/outcomes/estimation	Numbers analyzed, outcome results, effect estimate and precision	6–8	Partial – numbers analyzed, group results, p values and partial eta-squared are reported; confidence intervals/precision estimates are not reported.
27	Harms/results	Harms or unintended events in each group	6	Reported – no adverse events were reported in either group during the 12-week intervention.
28	Ancillary analyses	Other analyses, prespecified vs post hoc	5	Reported where applicable – TNF-α outlier analysis is described; no ancillary subgroup analyses are reported.
29	Interpretation	Interpretation consistent with results and relevant evidence	8–11	Reported.
30	Limitations	Bias, imprecision, generalizability and multiplicity as relevant	11	Reported – sample-size limitations and lack of control over diet and physical activity outside the program are discussed.
